# A putative nitroreductase from the DosR regulon of *Mycobacterium tuberculosis* induces pro-inflammatory cytokine expression *via* TLR2 signaling pathway

**DOI:** 10.1038/srep24535

**Published:** 2016-04-20

**Authors:** Vidyullatha Peddireddy, Sankara Narayana Doddam, Insaf A. Qureshi, Priyadarshini Yerra, Niyaz Ahmed

**Affiliations:** 1 Pathogen Biology Laboratory, Department of Biotechnology and Bioinformatics, University of Hyderabad, Hyderabad 500046 India; 2 Department of Biotechnology and Bioinformatics, University of Hyderabad, Hyderabad India

## Abstract

Tuberculosis caused by *Mycobacterium tuberculosis* is a global encumbrance and it is estimated that nearly one third population of the world acts as a reservoir for this pathogen without any symptoms. In this study, we attempted to characterise one of the genes of DosR regulon, Rv3131, a FMN binding nitroreductase domain containing protein, for its ability to alter cytokine profile, an essential feature of *M. tuberculosis* latency. Recombinant Rv3131 stimulated pro-inflammatory cytokines in THP-1 cells and human peripheral blood mononuclear cells in a time and dose dependent manner. *In silico* analyses using docking and simulations indicated that Rv3131 could strongly interact with TLR2 *via* a non-covalent bonding which was further confirmed using cell based colorimetric assay. In THP-1 cells treated with Rv3131 protein, a significant upsurge in the surface expression, overall induction and expression of mRNA of TLR2 was observed when analysed by flow cytometry, western blotting and real time PCR, respectively. Activation of TLR2 by Rv3131 resulted in the phosphorylation of NF- κβ. Results of this study indicate a strong immunogenic capability of Rv3131 elicited *via* the activation of TLR2 signalling pathway. Therefore, it can be surmised that cytokine secretion induced by Rv3131 might contribute to establishment of *M. tuberculosis* in the granulomas.

Tuberculosis remains a global burden and is accountable for elevated morbidity and mortality. Though individuals remain asymptomatic, in most cases *M. tuberculosis* remains in a latent stage. Then, the bacilli unleash their responses inside a specialised structure called granuloma; the microenvironment of the granulomas is characterized by hypoxia and elevated nitric oxide^1^. *M. tuberculosis* survives in this unreceptive *milieu* by up-regulating a set of 48 genes of the dormancy survival regulon (DosR), whose protein products regulate a variety of physiological processes of the bacterium. A hallmark of *M. tuberculosis* infection is the organism’s capability to rapidly acclimatise to altering environments since the cellular and biochemical dynamics of the granuloma are reliant on the oxygen tension^2^. Alternatively, during reactivation, *M. tuberculosis* adjusts to the aerobic conditions by switching on its metabolic activity. Under both these conditions, the DosR dependent regulation of DosR regulon member genes plays a critical role^3^. The protein products encoded by DosR genes can elicit humoral responses in the host, especially in patients with latent infection^4^. During latency, granuloma formation and its conservation requires production of pro-inflammatory cytokines and reactive oxygen intermediates^5^. These responses are instigated by the interaction of *M. tuberculosis* components with TLR1/TLR6 and TLR4 (Toll-like receptors) of macrophages^6^.

TLRs, also known as pattern recognition receptors are responsible for the detection of specific molecular components on the surface of the pathogens to induce immunologic responses. The cell wall components of *M. tuberculosis*, namely peptidoglycans, interact with TLR2 and influence expression of CD25, CD69, NKp44 and IFN-γ production in NK cells^7^. The lipid fractions of three different strains of *M. tuberculosis* elicited TLR2, cytokines and MHC class II expression in human macrophages^8^. Further, mycobacterial lipoprotein interaction results in TLR2 mediated apoptosis of T-cells^9^. Mycobacterial lipoarabinomannan mediated interaction between TLR1 and TLR2 has been reported^10^. Other TLRs on the macrophage surface were also found to be recognising mycobacterial proteins and lipids^11^. Such recognition results in the production of pro and anti-inflammatory cytokines, which in turn dictates macrophage effector functions^12^. A majority of the studies hitherto remained focused on the interaction of mycobacterial cell wall components and TLRs. However, such interactions between the proteins encoded by the DosR genes and TLRs are not very well studied.

Proteins encoded by the DosR regulon genes elicit immunomodulatory responses in the host. Some of the DosR antigens that were found to be immunodominant include alpha-cystallin 2 (Acr2; *Rv0251*)^13^, the alpha-crystallin homologue (also called 16-kDa protein; *Rv2031c*, *HspX*)^14^, Ag85A (*Rv3804*)^15^, *Hsp65 (Rv0440)*^16^, *ESAT-6* (*Rv3875*)^17^, and *CFP10* (*Rv3874*)^18^. Using mouse models and case controlled human studies in geographically diverse locations, the immunogenicity elicited by DosR regulon genes in latent infection was established^4^,^19^,^20^,^21^. Due to their immune-dominant nature they were considered to be potential candidates for the development of TB vaccines^22^. We previously reported and characterised the Dormancy Associated Translation Inhibitor (*Rv0079*/ DATIN) of *M. tuberculosis*, a protein coded by the DosR gene *Rv0079* and which was shown to be potentially immunogenic and leading to secretion of cytokines in macrophages and PBMCs; the immunomodulatory activity of DATIN was due to its interaction with TLR2^23^,^27^. Such interactions for other DosR antigens are not yet deciphered.

Rv3131, a hitherto uncharacterised member of the DosR regulon codes for a nitroreductase that might be involved in detoxification of nitrogen by-products in the host^24^. Nitroreductase genes, *acg* (*Rv2032*), *Rv3131* and *Rv3127* were predicted to guard against the nitrogen stress^25^. Rv3131, expressed robustly under stress, was found to contain two DevR/DosR binding sites^24^. The transcription of *Rv3131* encompasses binding of the DosR to the promoter in a cooperative manner^24^. Besides induction under hypoxic conditions, Rv3131 was also found to be antigenic^26^. In an earlier study comprising an African population, Rv3131 was found to be one of the most potent inducers of cytokine production among a set of seven classical and fifty one DosR regulon-encoded *M. tuberculosis* recombinant protein antigens^19^.

Though the immunogenic property of most of the proteins encoded by the DosR genes was characterised extensively, the mechanisms by which they elicit this response are not yet fully understood. It is possible that the modulation of immune responses may be different for each of them or certain set of DosR antigens may use a common mechanism of action. Thus, it is essential to understand the molecular mechanisms involved in the immunogenic properties of the DosR regulon antigens. Except for the induction under stress conditions and the antigenic property, information on the role of Rv3131 in mycobacterial latency, manipulation of host responses and the molecular machinery used for its action was not yet deciphered. Characterising the antigenic nature of this protein and its contribution to the survival of *M. tuberculosis* under latency might facilitate novel strategies that can be directed in boosting the host’s immune system or attenuation of survival of *M. tuberculosis* in the granulomas, respectively. In this study, we endeavoured to analyse the ability of recombinant Rv3131 to elicit cytokine response and its interaction with TLR2/4 and the ensuing downstream signalling.

## Methodology

### Molecular cloning, expression and purification of Rv3131 gene

*Rv3131* was cloned as described previously with slight modifications^27^. Briefly, *Rv3131* gene was amplified (supplementary information: Figure S1) using H37Rv DNA as a template and cloned into pET28a plasmid between BamHI and Hind III restriction enzyme sites. The plasmid was then transformed in to *E.coli* BL21 (DE3) and the recombinant protein was induced with 0.5 mM IPTG (Sigma Aldrich, USA) for 4 hours in LB broth containing Kanamycin 50 μg/ml. Protein extraction was carried out as described earlier^27^. The purity of recombinant Rv3131 (rRv3131) was checked by electrophoresing different fractions of the eluates on SDS gels. Rv3131 resolved as a single band and the purity of the protein as visualised was more than 95%. Endotoxin contamination was removed by incubating the protein with polymyxin-B agarose (Sigma, MO, USA). Limulus amebocyte lysate assay (Pierce™ LAL Chromogenic Endotoxin Quantitation Kit, ThermoFisher, USA) was used to measure the endotoxin content of the recombinant protein. Endotoxin contamination was undetectable in the protein fractions incubated with Polymyxin-B agarose.

### Quantification of cytokines from culture supernatants of THP-1 cells and PBMCs

Isolation of peripheral blood mononuclear cells (PBMCs) from human blood was performed using Ficoll (GE Healthcare, USA) gradient method and 1 million PBMCs were seeded into each well suspended in 1 ml of RPMI (Invitrogen Life technologies, CA, USA) with 10% Fetal Bovine Serum (FBS) (Invitrogen Life technologies, CA, USA) and incubated overnight. Blood samples for isolation of PBMCs were collected from volunteers who appeared healthy and reported no history of tuberculosis. The study was carried out in accordance with the Code of Ethics of the World Medical Association (Declaration of Helsinki) for experiments involving humans. The study was approved by Ethics Committee of University of Hyderabad. An educated and written informed consent was obtained from all the subjects before obtaining the blood samples. THP-1 cells obtained from National Centre for Cell Science, Pune, India, were seeded (approximately 0.2 million per well) in 24 well plate and treated overnight with 10 ng/ml Phorbol 12-myristate 13-acetate (PMA) (Sigma, MO, USA) to allow differentiation into macrophage-like phenotype. THP-1 cells / PBMCs were treated with various concentrations of recombinant Rv3131 protein (rRv3131) (10, 100 and 1000 ng/ml) or LPS (100 ng/ml; positive control) (Sigma, USA). Cells treated with Proteinase K digested rRv3131 were used as negative control. After the treatment, supernatants were collected at 24 and 48 hrs time points and stored in −80 °C. Quantification of pro-inflammatory cytokines such as IL-1β, IL-8, TNF-α, and IFN-γ were performed using Elisa- Set Go eBiosciences (CA, USA) kit following manufacturer’s instructions.

### Gene expression by reverse transcription PCR analysis

Differentiated THP-1 cells were treated with or without rRv3131 protein and total RNA was extracted using Trizol (Sigma, USA). 2 μg of DNase (sigma) treated RNA was converted into cDNA by SuperScriptIII reverse transcriptase (Invitrogen, USA) following recommendations of the manufacturer. Real-time PCR was carried out on a realplex machine (Eppendorf, Hamburg, Germany), using SYBR Green SuperMix (Clontech, USA) as described earlier^28^. Gene specific primers used in this study are mentioned in table 1. Mean fold changes in various cytokines were analysed using the ΔΔCT method. Peptidylprolyl isomerase B (PPIB) and Glyceraldehyde-3-Phosphate Dehydrogenase (GAPDH) were used as housekeeping genes for cytokines and TLR2 / TLR4 respectively.

### Protein sequence analysis of Rv3131 and docking study with TLR2

Amino acid sequence of Rv3131 (332 residues) was retrieved from *Mycobacterium tuberculosis* Database (http://tuberculist.epfl.ch/) and subjected to Pfam^29^ and TOPCONS^30^ for sequence analysis. The program BLAST-P has been used to detect similar crystallographic protein structures of Rv3131 and the template structures were formerly downloaded from RCSB Protein Data Bank (PDB). Nitroreductase proteins from *Mycobacterium smegmatis* (PDB ID: 2YMV) and *Streptococcus pneumonia* (PDB ID: 2B67) were used as templates to perform homology modelling using Modeller9.15^31^. Further, the geometry of generated model was checked and analysed using PROCHECK^32^. PyMOL program was used for molecular visualization and MetaPPISP^33^ program was employed to predict the protein–protein interaction site. Simultaneously, for protein–protein docking studies, the web version of PatchDock^34^ was executed and further improvement and ranking was done using FireDock^35^. Docking studies were done under default complex-type settings using Rv3131 model (ligand) and the crystal structure of TLR2 (PDB ID: 2Z82) was obtained from Protein Data Bank (receptor).

### TLR2/TLR4 interaction assay

Interaction of Rv3131 with TLR2/TLR4 was analysed using HEK-Blue 293 hTLR-2 and hTLR-4 engineered cell lines (HEK-Blue™-hTLR-2/hTLR-4, InvivoGen, San Diego, US) which are co-transfected with TLR2/TLR4 and SEAP reporter gene (secreted embryonic alkaline phosphatase). Stimulation of TLR2/TLR4 ligands activates NF-κB and AP-1 which in turn induces the production of SEAP that can be readily assessed with HEK-Blue™ detection medium (with colour change from pink to purple) by measuring spectrophotometrically^36^. Different concentrations of rRv3131 (10–200 ng) was added to approximately 50,000 HEK-Blue 293 hTLR2 and 25,000 HEK-Blue 293 hTLR4 cells respectively. Another His-tagged recombinant protein, Rab5 was used as negative control and *Mycoplasma salivarium* derived synthetic lipoprotein FSL-1 (10 ng) (Sigma, USA) and LPS (100 ng) were used as positive controls. The intensity of colour formation was measured in an ELISA plate reader (Infinite^®^ 200 PRO - Tecan) at 620 nm.

### Flow Cytometry analysis

Surface expression of TLR2/TLR4 was determined by using differentiated THP-1 cells treated with various concentrations of rRv3131. Cells were harvested after a period of 24 hrs and incubated with goat anti-human TLR2 (2.5 μg/million cells) and goat anti-human TLR4 (R&D, MN, USA) or isotype-matched anti-goat mouse IgG antibodies for 60 min at 4 °C followed by incubation with donkey anti-goat IgG conjugated with FITC (0.5 μg/million cells) (Santa cruz, CA, USA) at 4 °C for another 45 min. Finally, cells were washed and re-suspended in 1× PBS containing 2% FBS. Fluorescence intensity was measured using BD FACS Canto II flow cytometer (BD Biosciences, NJ, USA). Untreated and stained cells were taken as negative control in both the assays. The data were analysed by Flow Jo software (Tree Star Inc., USA).

### Western blot analysis

The total protein was extracted from rRv3131 (1000 ng/ml) treated and untreated THP-1 cells. Trichostatin A (TSA) (250 ng) and LPS (100 ng/ml) were included as positive controls for checking TLR2 and NF-κβ activities, respectively. Untreated cells served as negative control. In brief, 1 × 10^6^ (TLR2) or 6 × 10^6^ (NF-κβ) cells were washed with cold 1× PBS and pelleted by centrifugation at 400 g for 5 min. Cell pellets were treated with ice cold lysis buffer (0.1% NP-40 in 2 mM EDTA, 200 mM Tris–HCl, pH, 7.5, 250 mM NaCl, 100 mM PMSF, 1 M DTT and protease inhibitor cocktail (Sigma, USA) by repeated pipetting at 4 °C. Supernatants, were collected after centrifugation at 13,000 g for 5 min and protein concentrations were analysed. Equal amounts of proteins were loaded and transferred on to a PVDF membrane at 4 °C. Subsequently, the membranes blocked with 4% BSA in TBST (137 mM NaCl, 20 mM Tris pH 7.6, and 0.05% Tween-20) for 3 h at room temperature and later incubated with primary antibodies against TLR2, p65 (NF-κβ) and β-actin antibodies (Santa Cruz Biotechnology Inc., USA). PVDF membranes were perceived by chemiluminescence (ECL). The band intensity in TLR2 assay was quantified densitometrically by Image J software^37^ and normalized to β-actin.

### Statistical analysis

For all the experiments, wherever required, Student’s t-test and one way ANOVA was executed for the analysis of the results. The data were represented as the mean of triplicates ± SEM. p < 0.05 was considered as significant. Data analysis was carried out using GraphPad Prism 5 software.

## Results

### Rv3131 induces pro-inflammatory cytokines

**Figure 1:**
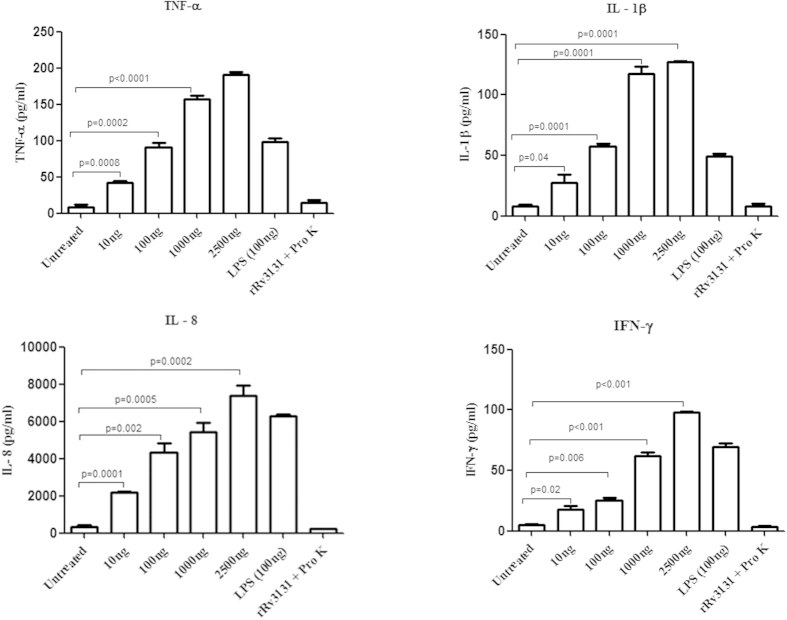
Rv3131 stimulates the secretion of pro-inflammatory cytokines in PBMCs. Isolated PBMCs from human blood were treated with 10–2500 ng/ml of rRv3131 or LPS (100 ng/ml) or proteinase K digested Rv3131 protein for 24 h. The levels of secreted cytokines like IL-1β, IFN-γ, TNF-α and IL-8 were quantified by ELISA. Data represent the mean ± SEM of three experimental replicates.

**Figure 2:**
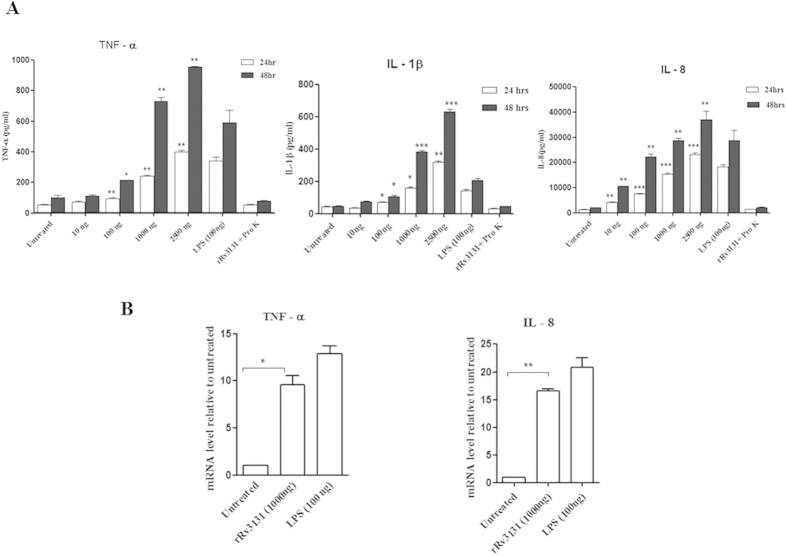
(**A**) Rv3131 stimulates the secretion of pro-inflammatory cytokines in THP-1 cells. THP-1 cells differentiated by PMA were stimulated with 10–2500 ng/ml of rRv3131 or LPS (100 ng/ml) or proteinase K digested rRv3131 for 24 and 48 h. Supernatants were collected and the levels of secreted cytokines were estimated by ELISA. Data represent the mean ± SEM of three technical replicates. *p < 0.05; **p < 0.005; ***p < 0.0005. (**B**) Cytokine gene expression: relative expression of TNF-α and IL-8 in THP-1 cell lines treated with rRv3131 (1000 ng) and LPS (100 ng) was evaluated by quantitative real time-PCR (qRT-PCR). PPIB was used as endogenous control for normalization. The data represent mean ± SEM of triplicates.

To determine whether recombinant Rv3131can induce pro-inflammatory responses, THP-1 cells and human PBMCs were treated with varying concentrations of this protein and the secreted cytokines (IL-1β, IFN-γ, TNF-α and IL-8) were measured in the supernatants. The positive control, LPS (100 ng) induced the production of these cytokines at similar conditions tested. Treatment of PBMCs with rRv3131 resulted in increased production of IFN-γ, TNF-α, IL-1 β and IL-8 in a dose dependent manner (Fig. 1). Similarly in the THP-1 cells rRv3131 was observed to induce TNF-α, IL-1 β and IL-8 production in a time and dose dependent manner (Fig. 2A). The negative control (rRv3131 digested with proteinase K) did not affect the cytokine production. In order to analyse whether the increased production of pro-inflammatory cytokines in THP-1 cells after rRv3131 treatment was due to modulation at the transcriptional level, real-time PCR was performed. Significant increase in the mRNA levels of IL-8 and TNF-α was observed (Fig. 2B).

### Protein sequence analysis of Rv3131 and docking study with TLR2

**Figure 3:**
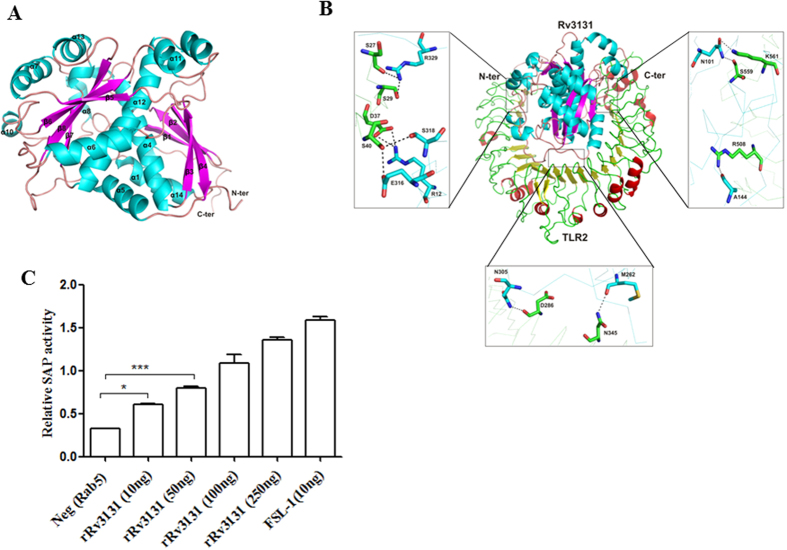
Modelled 3D-structure of Rv3131 and its interaction with TLR2. (**A**) Three dimensional structure of Rv3131 determined using homology modelling: the elements of protein secondary structure were coloured and labelled (helices and sheets displayed in cyan and pink, respectively). (**B**) Docking study of Rv3131 with TLR2: the residues of TLR2 and Rv3131 are coloured in green and cyan, respectively. The residues presenting interaction among both the proteins are labelled and shown as stick model in element colours (green/cyan colour represents carbon, blue represents nitrogen, and red represents oxygen). The black dashed lines represent hydrogen bonds. (**C**) Rv3131 interacts with TLR2: HEK-Blue 293 hTLR-2 cells were treated with various concentrations (10 ng–250 ng) of rRv3131 and interaction of Rv3131 with TLR2 was measured spectrophotometrically by the colour change read at 620 nm due to the activity of SEAP. *p < 0.05; ***p < 0.0005.

Computational analysis demonstrated that Rv3131 possessed conserved nitroreductase domain spanning 236–301 residues, while TOPCONS suggested the lack of signal peptide or transmembrane region in this protein. As crystallographic/solution structure of this protein is not available, BLASTp was used to find the structural templates and a template (PDB ID: 2YMV, nitroreductase from *Mycobacterium smegmatis*) with 37% identity was retrieved from PDB data bank. Modeller was used to generate structures using single template and the best structure was selected on the basis of different parameters including low DOPE score. To analyse the quality of structure, Ramachandran plot was generated and it revealed two residues (L327 and L328) in disallowed region. To improve quality of the structure, modelling was performed further with two templates i.e. PDB ID: 2YMV and 2B67. The quality of the structure was evaluated using Ramachandran plot acquired *via* Procheck and results of the validated model were as follows: most favoured regions contained 89.9%; additional allowed regions had 9.4%; generously allowed regions had 0.7% and disallowed regions contained 0.0% residues. Secondary structure of modelled Rv3131 protein revealed fourteen alpha helices and eight beta sheets (Fig. 3A). Identification of possible binding interface residues was performed using MetaPPISP programme. Protein-protein docking was performed with receptor as TLR2 and ligand as Rv3131 model using different online servers including GRAMM-X^38^ and PatchDock. Among the used programmes, Patchdock provided several solutions based on shape complementarity criteria, where Rv3131 was placed besides TLR2. Out of obtained complexes, best 10 were further subjected to FireDock to refine the solutions based on global energy. However, complex 7 was evolved as the best solution for residues interaction between the two proteins. As shown in Fig. 3B, Rv3131 emanates close to TLR2 and shows strong interaction due to the presence of several non-covalent bonds between them.

### Rv3131 interacts with TLR2

To lend further credence to the *in silico* observations, the interaction between Rv3131 and TLR2 was analysed using a cell based assay. In HEK-Blue 293 hTLR2 cells expressing TLR2 on their cell surface, alkaline phosphatase activity was found to be augmented with the increase in rRv3131 treatment. Similar increase in activity was observed in cells treated with the positive control FSL-1. The negative control, Rab5 did not cause any increase in alkaline phosphatase activity, thus validating the assay (Fig. 3C). Such an increase in activity was not observed in HEK-Blue 293 hTLR-4 cells expressing TLR4 on their cell surface when treated with Rv3131 protein, suggesting that the interaction of this protein was specific to TLR2 (data not shown).

### Rv3131 induces TLR2 expression

**Figure 4:**
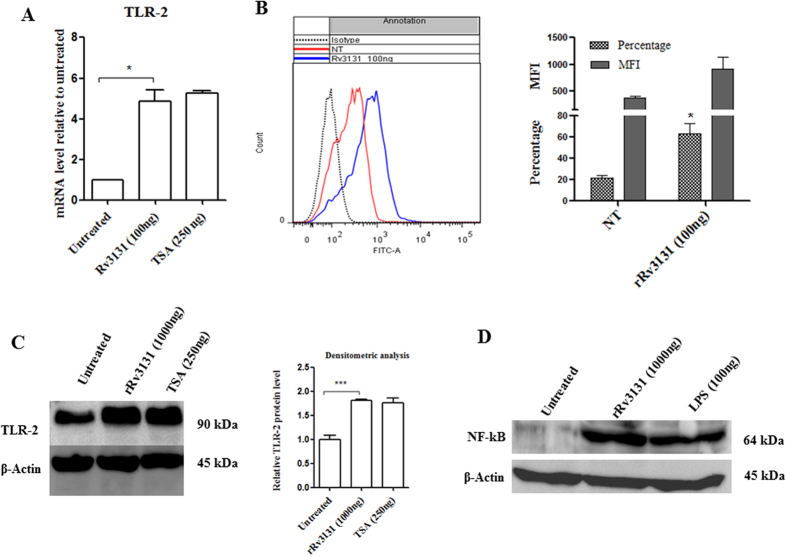
Rv3131 induces TLR2 expression and NF-κB activation. (**A**) Real time expression of TLR2: RNA isolated from differentiated THP-1 cells treated with rRv3131 (1000 ng) or TSA (250 ng) was reverse transcribed and real time PCR was carried out using gene specific primers. The expression of glyceraldehyde 3-phosphate dehydrogenase (*GAPDH*) was used as internal control. Data are presented as mean ± SEM of three independent experiments. (**B**) Surface expression of TLR2 by flow cytometry: PMA-differentiated THP-1 cells were treated with 100 ng of rRv3131 for 24 h followed by incubation with primary and secondary FITC labelled antibodies and analysed by flow cytometry. Data are expressed as mean ± SEM of percentage of cell population/MFI values from three independent experiments. (**C**) TLR2 protein expression: total protein isolated from differentiated THP-1 cells treated with rRv3131 (1000 ng) or LPS (100 ng) was electrophoresed on polyacrylamide gel, transferred onto PVDF membrane and probed with antibodies against TLR2 and β-actin (internal control). Signal corresponding to the intensity of the band was measured using chemiluminiscence. The graph represents relative TLR2 expression levels quantified densitometrically (**D**) NF-κB phosphorylation: the total protein isolated was analysed using western blot with antibodies specific to phosphorylated p65.

The possible role of Rv3131 in modulating TLR2 expression, besides its interaction on the cell surface was analysed by real time PCR. In THP-1 cells treated with rRv3131 protein, TLR2 mRNA levels were significantly upregulated (Fig. 4A) similar to that of the positive control TSA. Besides the gene expression, TLR2 expression levels on THP-1 cell surface following rRv3131 treatment were also analysed using flow cytometry analysis. A significant increase in the expression of TLR2 (Fig. 4B) but not TLR4 (data not shown) was observed in THP-1 cells when treated with rRv3131. Western blot analyses also indicated an increased expression of TLR2 in rRv3131 treated THP-1 cells (Fig. 4C). To determine whether the interaction of Rv3131 with TLR2 initiates NF-κB signalling pathway, western blot was performed. Analysis of whole cell protein isolated from THP-1 cells revealed activated form of NF- κB (p65) in rRv3131 and LPS treated preparations, but not in untreated cells (Fig. 4D).

## Discussion

Despite existence of tuberculosis infection worldwide, since ancient times, its pathology remained complicated even after extensive research. Majority of the tuberculosis patients experience latent phase infection before the active disease sets in. During this phase, immune responses to latency associated antigens are detected. The existing treatment approaches turn out to be ineffective due to a unique ability of this pathogen to remain dormant by vesting in granulomas, impermeable to the anti-tuberculosis drugs^39^. Adding to this is the lack of accurate *in vitro* or *in vivo* models that can mimic *M. tuberculosis* latency and/or resuscitation. Thus, development of effective vaccines using *M. tuberculosis* antigens is essential. Though classical antigens of *M. tuberculosis* were proposed to be potential vaccine candidates, because of the complicated life cycle of this pathogen, which includes latency, developing vaccines using antigens that are specifically expressed in latent stage is gaining prominence^40^. Recent studies identified that genes of DosR, upregulated during latency, also serve as antigens eliciting immune response^4^,^41^. Rv3131 was demonstrated to be significantly upregulated (up to 40 folds) under *in vitro* dormancy conditions^21^. Thus, such antigens were projected to be potential vaccine candidates due to their stage specific expression and immunogenicity.

We observed increased pro-inflammatory cytokine induction in THP-1 cells and human PBMCs with the treatment of recombinant rRv3131. Though a dose dependent increase in cytokine levels was observed in THP-1 cells and PBMCs treated with Rv3131, the highest dose (2500 ng) did not augment the release of IL-1β over 100 ng. This variation in IL-1β response could be due to physiological and phenotypic differences between the two cell types. The ability of Rv3131 to elicit immune response in *M. tuberculosis* specific T-cells obtained from tuberculin skin test positive patients was demonstrated^4^. In an Ugandan population, Rv3131 was demonstrated to be one of the most immunogenic antigens by virtue of its ability to induce interferon-γ^19^. TNF-α levels were found to be high in the lungs of mice with persistent tuberculosis^42^ and is known to contribute to many immune regulatory functions such as macrophage activation and granuloma formation^5^,^43^. The ability of Rv3131 to induce pro-inflammatory cytokines suggests that it might activate macrophages during latency to govern the micro environment of the granuloma and favor the survival of latent *M. tuberculosis.* Recognition of *M. tuberculosis* in the host is executed by TLRs, especially TLR2 and to some extent by TLR4. The role of TLR2 in mycobacterial recognition and the downstream signalling during *M. tuberculosis* infection was well proven in a variety of TLR knock out animal models^44^ and the susceptibility observed due to genetic polymorphisms in their genes^45^ was demonstrated. Interaction of mycobacterial products with TLR2 leads to the secretion of pro-inflammatory^23^,^46^ and anti-inflammatory cytokines^47^. It should be noted that *M. tuberculosis* is an intracellular pathogen and the proteins secreted by it could also enter or be located in extracellular spaces during infections. We earlier characterized the mycobacterial Hsp65 and found it to have dual roles in autoimmunity and inflammation^48^. It is possible that Rv3131 could also reach the extracellular space and presented to immune cells to trigger cytokine secretion. Though our study proves that cytokine secretion induced by Rv3131 involves TLR2 and NF- κB, the possibility that cytokine secretion is also due to presentation of Rv3131 to immune cells cannot be ruled out. It is worthwhile to mention that cytokine response in isolated PBMCs could also be contributed by other cells such as dendritic cells, T and B cells, etc. Analysing cytokine expression using purified fractions of each of these cells would provide information on the individual cell type contribution and/or cell specificity, if any, in Rv3131 induced cytokine response.

Interaction of mycobacterial classical antigens such as peptidoglycan, with TLR2 marks the commencement of downstream signaling^7^. We previously demonstrated that the DosR regulon antigen DATIN can interact and activate TLR2 resulting in secretion of pro inflammatory cytokines^23^. Similarly, PE35 (*Rv3872*), a mycobacterial protein physically interacts with TLR2 resulting in a dose dependent increase in the secretion of pro-inflammatory cytokine secretion^49^. The interaction of Rv3131 predicted in this study was based on computational modeling that generates models with a high degree of confidence. However, detailed analyses of the binding pockets and the associated specificity need to be addressed using site directed mutagenesis of both the ligand and the receptor.

In the current study, besides interacting with TLR2, Rv3131 induced TLR2 mRNA and protein expression. Modulation of TLR2 expression by the classical *M. tuberculosis* antigens was reported^50^. For example, the lipid fractions of three different strains of *M. tuberculosis* had differential effects on the expression profile of TLR2 and TLR4 cytokines and MHC class II components^8^. Another study indicated that the ESAT-6 antigen of *M. tuberculosis* modulates T helper cell responses *via* TLR-2^51^. Further, in our studies, phosphorylation of NF- κB was evident in macrophages treated with recombinant Rv3131. Activation of NF-κB in the macrophages by the mycobacteria has been reported^52^. Given this, it is possible that Rv3131 might modulate the expression of TLR2 and may interact with it to influence the downstream signaling *via* NF-κB leading to the secretion of cytokines that favor the survival of *M. tuberculosis* in the granulomas. We used an *in vitro* system to study the immunogenic properties of Rv3131 protein at varying concentrations. The physiological concentration of Rv3131 in the granuloma during latency is very difficult to predict or estimate. However, it is an accepted practice to analyze the immunogenic properties of antigens using *in vitro* models. Hence, the results presented in our study strongly support that Rv3131 might induce pro-inflammatory cytokine expression *via* TLR2 signaling pathway and give an impetus to the understanding of the molecular mechanisms of regulation of DosR antigens.

In conclusion, we report that the DosR antigen, Rv3131 may possibly influence the innate immune responses to facilitate the survival of *M. tuberculosis* in the granulomatous microenvironment. This is brought about by induction of cytokine secretion mediated by its interaction with TLR2. Given paucity of knowledge on the mechanisms by which DosR antigens influence the *milieu* of the granuloma and support *M. tuberculosis* survival in a latent state, results of this study contribute suggestively to the understanding of the survival of *M. tuberculosis* executed *via* its latency specific antigens.

## Additional Information

**How to cite this article**: Peddireddy, V. *et al.* A putative nitroreductase from the DosR regulon of *Mycobacterium tuberculosis* induces pro-inflammatory cytokine expression via TLR2 signaling pathway. *Sci. Rep.* **6**, 24535; doi: 10.1038/srep24535 (2016).

## Supplementary Material

Supplementary Information

## Figures and Tables

**Table 1:** List of PCR and qRT-PCR primers.

S.No	Gene Name	Primer Sequence	Ref.
1	*Rv3131*	F-5′-CG**GGATCC**ATGAACACCCATTTCCCG-3′	This study
		R- 5′-CC**AAGCTT**TCAGCACCGTTGTCGCAG-3′	
2	*TNF - α*	F-5′-TTC TCC TTC CTG ATC GTG GC-3′	28
		R-5′-ACT CGG GGT TCG AGA AGA TG-3′	
3	*IL - 8*	F-5′-CTG GCC GTG GCT CTC TTG-3′	53
		R-5′-CCT TGG CAA AAC TGC ACC TT-3′	
4	*Peptidylprolyl isomerase-B (PPIB)*	F-5′-ATG TAG GCC GGG TGA TCT TT-3′	28
		R-5′-TGA AGT TCT CAT CGG GGA AG-3′	
5	*TLR - 2*	F-5′-GGC CAG CAA ATT ACC TGT GTG-3′	53
		R-5′-AGG CGG ACA TCC TGA ACC T-3′	
6	*TLR - 4*	F-5′-CTG CAA TGG ATC AAG GAC CA -3′	53
		R-5′-TTA TCT GAA GGT GTT GCA CAT TCC-3′	
7	*Glyceraldehyde-3-Phosphate Dehydrogenase (GAPDH)*	F-5′-GGA AGG TGA AGG TCG GAG TC-3′	54
		R-5′-TGA GGT CAA TGA AGG GGT CA-3′	

## References

[b1] Forrellad M. A. *et al.* Virulence factors of the Mycobacterium tuberculosis complex. Virulence 4, 3–66 (2013). 23076359 10.4161/viru.22329 PMC3544749

[b2] Guirado E. *et al.* Characterization of host and microbial determinants in individuals with latent tuberculosis infection using a human granuloma model. MBio 6, e02537–02514 (2015). 25691598 10.1128/mBio.02537-14 PMC4337582

[b3] Watanabe S. *et al.* Fumarate reductase activity maintains an energized membrane in anaerobic Mycobacterium tuberculosis. PLoS Pathog 7, e1002287 (2011). 21998585 10.1371/journal.ppat.1002287 PMC3188519

[b4] Leyten E. M. *et al.* Human T-cell responses to 25 novel antigens encoded by genes of the dormancy regulon of Mycobacterium tuberculosis. Microbes Infect 8, 2052–2060 (2006). 16931093 10.1016/j.micinf.2006.03.018

[b5] Sasindran S. J. & Torrelles J. B. Mycobacterium Tuberculosis Infection and Inflammation: what is Beneficial for the Host and for the Bacterium? Front Microbiol 2, 2 (2011). 21687401 10.3389/fmicb.2011.00002 PMC3109289

[b6] Basu J., Shin D. M. & Jo E. K. Mycobacterial signaling through toll-like receptors. Front Cell Infect Microbiol 2, 145 (2012). 23189273 10.3389/fcimb.2012.00145 PMC3504976

[b7] Esin S. *et al.* Interaction of Mycobacterium tuberculosis cell wall components with the human natural killer cell receptors NKp44 and Toll-like receptor 2. Scand J Immunol 77, 460–469 (2013). 23578092 10.1111/sji.12052

[b8] Rocha-Ramirez L. M. *et al.* Mycobacterium tuberculosis lipids regulate cytokines, TLR-2/4 and MHC class II expression in human macrophages. Tuberculosis (Edinb) 88, 212–220 (2008). 18222732 10.1016/j.tube.2007.10.003

[b9] Bocchino M. *et al.* Role of mycobacteria-induced monocyte/macrophage apoptosis in the pathogenesis of human tuberculosis. Int J Tuberc Lung Dis 9, 375–383 (2005). 15830742

[b10] Tapping R. I. & Tobias P. S. Mycobacterial lipoarabinomannan mediates physical interactions between TLR1 and TLR2 to induce signaling. J Endotoxin Res 9, 264–268 (2003). 12935358 10.1179/096805103225001477

[b11] Jo E. K., Yang C. S., Choi C. H. & Harding C. V. Intracellular signalling cascades regulating innate immune responses to Mycobacteria: branching out from Toll-like receptors. Cell Microbiol 9, 1087–1098 (2007). 17359235 10.1111/j.1462-5822.2007.00914.x

[b12] Collins H. L. & Kaufmann S. H. The many faces of host responses to tuberculosis. Immunology 103, 1–9 (2001). 11380686 10.1046/j.1365-2567.2001.01236.x PMC1783212

[b13] Wilkinson K. A. *et al.* Infection biology of a novel alpha-crystallin of Mycobacterium tuberculosis: Acr2. J Immunol 174, 4237–4243 (2005). 15778386 10.4049/jimmunol.174.7.4237

[b14] Geluk A. *et al.* T-cell recognition of the HspX protein of Mycobacterium tuberculosis correlates with latent M. tuberculosis infection but not with M. bovis BCG vaccination. Infect Immun 75, 2914–2921 (2007). 17387166 10.1128/IAI.01990-06 PMC1932904

[b15] Smith S. M. *et al.* Human CD8+ CTL specific for the mycobacterial major secreted antigen 85A. J Immunol 165, 7088–7095 (2000). 11120838 10.4049/jimmunol.165.12.7088

[b16] Ottenhoff T. H., Ab B. K., Van Embden J. D., Thole J. E. & Kiessling R. The recombinant 65-kD heat shock protein of Mycobacterium bovis Bacillus Calmette-Guerin/M. tuberculosis is a target molecule for CD4+ cytotoxic T lymphocytes that lyse human monocytes. J Exp Med 168, 1947–1952 (1988). 2903217 10.1084/jem.168.5.1947 PMC2189100

[b17] Demissie A. *et al.* Recognition of stage-specific mycobacterial antigens differentiates between acute and latent infections with Mycobacterium tuberculosis. Clin Vaccine Immunol 13, 179–186 (2006). 16467323 10.1128/CVI.13.2.179-186.2006 PMC1391929

[b18] Skjot R. L. *et al.* Comparative evaluation of low-molecular-mass proteins from Mycobacterium tuberculosis identifies members of the ESAT-6 family as immunodominant T-cell antigens. Infect Immun 68, 214–220 (2000). 10603390 10.1128/iai.68.1.214-220.2000 PMC97123

[b19] Black G. F. *et al.* Immunogenicity of novel DosR regulon-encoded candidate antigens of Mycobacterium tuberculosis in three high-burden populations in Africa. Clin Vaccine Immunol 16, 1203–1212 (2009). 19553548 10.1128/CVI.00111-09 PMC2725533

[b20] Timm J. *et al.* Differential expression of iron-, carbon-, and oxygen-responsive mycobacterial genes in the lungs of chronically infected mice and tuberculosis patients. Proc Natl Acad Sci USA 100, 14321–14326 (2003). 14623960 10.1073/pnas.2436197100 PMC283590

[b21] Voskuil M. I. *et al.* Inhibition of respiration by nitric oxide induces a Mycobacterium tuberculosis dormancy program. J Exp Med 198, 705–713 (2003). 12953092 10.1084/jem.20030205 PMC2194188

[b22] Sander C. & McShane H. Translational mini-review series on vaccines: Development and evaluation of improved vaccines against tuberculosis. Clin Exp Immunol 147, 401–411 (2007). 17302888 10.1111/j.1365-2249.2006.03306.x PMC1810501

[b23] Kumar A. *et al.* Dormancy Associated Translation Inhibitor (DATIN/Rv0079) of Mycobacterium tuberculosis interacts with TLR2 and induces proinflammatory cytokine expression. Cytokine 64, 258–264 (2013). 23819907 10.1016/j.cyto.2013.06.310

[b24] Chauhan S. & Tyagi J. S. Powerful induction of divergent tgs1-Rv3131 genes in Mycobacterium tuberculosis is mediated by DevR interaction with a high-affinity site and an adjacent cryptic low-affinity site. J Bacteriol 191, 6075–6081 (2009). 19648251 10.1128/JB.00310-09 PMC2747894

[b25] Hu Y. & Coates A. R. Mycobacterium tuberculosis acg gene is required for growth and virulence *in vivo*. PLoS One 6, e20958 (2011). 21687631 10.1371/journal.pone.0020958 PMC3110807

[b26] Riano F. *et al.* T cell responses to DosR and Rpf proteins in actively and latently infected individuals from Colombia. Tuberculosis (Edinb) 92, 148–159 (2012). 22226907 10.1016/j.tube.2011.12.005

[b27] Kumar A. *et al.* Mycobacterium tuberculosis DosR regulon gene Rv0079 encodes a putative, ‘dormancy associated translation inhibitor (DATIN)’. PLoS One 7, e38709 (2012). 22719925 10.1371/journal.pone.0038709 PMC3374827

[b28] Devi S., Rajakumara E. & Ahmed N. Induction of Mincle by Helicobacter pylori and consequent anti-inflammatory signaling denote a bacterial survival strategy. Sci Rep 5, 150499 (2015). 10.1038/srep15049 PMC4601021 26456705

[b29] Finn R. D. *et al.* Pfam: the protein families database. Nucleic Acids Res 42, D222–230 (2014). 24288371 10.1093/nar/gkt1223 PMC3965110

[b30] Tsirigos K. D., Peters C., Shu N., Kall L. & Elofsson A. The TOPCONS web server for consensus prediction of membrane protein topology and signal peptides. Nucleic Acids Res 43, W401–407 (2015). 25969446 10.1093/nar/gkv485 PMC4489233

[b31] Webb B. & Sali A. Comparative Protein Structure Modeling Using MODELLER. Curr Protoc Bioinformatics 47, 561–5632 (2014). 10.1002/0471250953.bi0506s47 25199792

[b32] Laskowski R. A., Chistyakov V. V. & Thornton J. M. PDBsum more: new summaries and analyses of the known 3D structures of proteins and nucleic acids. Nucleic Acids Res 33, D266–268 (2005). 15608193 10.1093/nar/gki001 PMC539955

[b33] Qin S. & Zhou H. X. meta-PPISP: a meta web server for protein-protein interaction site prediction. Bioinformatics 23, 3386–3387 (2007). 17895276 10.1093/bioinformatics/btm434

[b34] Schneidman-Duhovny D., Inbar Y., Nussinov R. & Wolfson H. J. PatchDock and SymmDock: servers for rigid and symmetric docking. Nucleic Acids Res 33, W363–367 (2005). 15980490 10.1093/nar/gki481 PMC1160241

[b35] Mashiach E., Schneidman-Duhovny D., Andrusier N., Nussinov R. & Wolfson H. J. FireDock: a web server for fast interaction refinement in molecular docking. Nucleic Acids Res 36, W229–232 (2008). 18424796 10.1093/nar/gkn186 PMC2447790

[b36] Konieczna P. *et al.* Bifidobacterium infantis 35624 administration induces Foxp3 T regulatory cells in human peripheral blood: potential role for myeloid and plasmacytoid dendritic cells. Gut 61, 354–366 (2012). 22052061 10.1136/gutjnl-2011-300936

[b37] Schneider C. A., Rasband W. S. & Eliceiri K. W. NIH Image to ImageJ: 25 years of image analysis. Nat Methods 9, 671–675 (2012). 22930834 10.1038/nmeth.2089 PMC5554542

[b38] Tovchigrechko A. & Vakser I. A. GRAMM-X public web server for protein-protein docking. Nucleic Acids Res 34, W310–314 (2006). 16845016 10.1093/nar/gkl206 PMC1538913

[b39] Smith T., Wolff K. A. & Nguyen L. Molecular biology of drug resistance in Mycobacterium tuberculosis. Curr Top Microbiol Immunol 374, 53–80 (2013). 23179675 10.1007/82_2012_279 PMC3982203

[b40] Kaufmann S. H. The contribution of immunology to the rational design of novel antibacterial vaccines. Nat Rev Microbiol 5, 491–504 (2007). 17558425 10.1038/nrmicro1688

[b41] Serra-Vidal M. M. *et al.* Immunogenicity of 60 novel latency-related antigens of Mycobacterium tuberculosis. Front Microbiol 5, 517 (2014). 25339944 10.3389/fmicb.2014.00517 PMC4189613

[b42] Flynn J. L., Scanga C. A., Tanaka K. E. & Chan J. Effects of aminoguanidine on latent murine tuberculosis. J Immunol 160, 1796–1803 (1998). 9469439

[b43] Cooper A. M. Cell-mediated immune responses in tuberculosis. Annu Rev Immunol 27, 393–422 (2009). 19302046 10.1146/annurev.immunol.021908.132703 PMC4298253

[b44] Noss E. H. *et al.* Toll-like receptor 2-dependent inhibition of macrophage class II MHC expression and antigen processing by 19-kDa lipoprotein of Mycobacterium tuberculosis. J Immunol 167, 910–918 (2001). 11441098 10.4049/jimmunol.167.2.910

[b45] Thada S., Valluri V. L. & Gaddam S. L. Influence of Toll-like receptor gene polymorphisms to tuberculosis susceptibility in humans. Scand J Immunol 78, 221–229 (2013). 23672492 10.1111/sji.12066

[b46] Basu S. *et al.* Execution of macrophage apoptosis by PE_PGRS33 of Mycobacterium tuberculosis is mediated by Toll-like receptor 2-dependent release of tumor necrosis factor-alpha. J Biol Chem 282, 1039–1050 (2007). 17095513 10.1074/jbc.M604379200

[b47] Nair S. *et al.* The PPE18 of Mycobacterium tuberculosis interacts with TLR2 and activates IL-10 induction in macrophage. J Immunol 183, 6269–6281 (2009). 19880448 10.4049/jimmunol.0901367

[b48] Rani P. S. *et al.* Mycobacterial Hsp65 potentially cross-reacts with autoantibodies of diabetes sera and also induces (*in vitro*) cytokine responses relevant to diabetes mellitus. Mol Biosyst 9, 2932–2941 (2013). 24056978 10.1039/c3mb70307j

[b49] Tiwari B., Soory A. & Raghunand T. R. An immunomodulatory role for the Mycobacterium tuberculosis region of difference 1 locus proteins PE35 (Rv3872) and PPE68 (Rv3873). FEBS J 281, 1556–1570 (2014). 24467650 10.1111/febs.12723

[b50] Harding C. V. & Boom W. H. Regulation of antigen presentation by Mycobacterium tuberculosis: a role for Toll-like receptors. Nat Rev Microbiol 8, 296–307 (2010). 20234378 10.1038/nrmicro2321 PMC3037727

[b51] Chatterjee S. *et al.* Early secreted antigen ESAT-6 of Mycobacterium tuberculosis promotes protective T helper 17 cell responses in a toll-like receptor-2-dependent manner. PLoS Pathog 7, e1002378 (2011). 22102818 10.1371/journal.ppat.1002378 PMC3213116

[b52] Kumar P., Tyagi R., Das G. & Bhaskar S. Mycobacterium indicus pranii and Mycobacterium bovis BCG lead to differential macrophage activation in Toll-like receptor-dependent manner. Immunology 143, 258–268 (2014). 24766519 10.1111/imm.12306 PMC4172141

[b53] Hayashi F., Means T. K. & Luster A. D. Toll-like receptors stimulate human neutrophil function. Blood 102, 2660–2669 (2003). 12829592 10.1182/blood-2003-04-1078

[b54] Moore X. L. *et al.* Endothelial progenitor cells’ “homing” specificity to brain tumors. Gene Ther 11, 811–818 (2004). 15057261 10.1038/sj.gt.3302151

