# Supplementary material for: A putative nitroreductase from the DosR regulon of Mycobacterium tuberculosis induces pro-inflammatory cytokine expression via TLR2 signaling pathway
Source: Sci Rep. 2016 Apr 20;6:24535. doi: 10.1038/srep24535 (PMC4837367; doi:10.1038/srep24535)
Supplement: Supplementary Information [file srep24535-s1.pdf]

Supplementary Information

A putative nitroreductase from the DosR regulon of *Mycobacterium tuberculosis* induces pro-inflammatory cytokine expression *via* TLR2 signaling pathway

Vidyullatha Peddireddy [1], Sankara Narayana Doddam[1], Insaf A. Qureshi[2], Priyadarshini Yerra [1] and Niyaz Ahmed[1 *]

1. Pathogen Biology Laboratory, Department of Biotechnology and Bioinformatics, University of Hyderabad, Hyderabad, India.
2. Department of Biotechnology and Bioinformatics, University of Hyderabad, Hyderabad, India

This supplementary file contains Figure S1 and its legend.

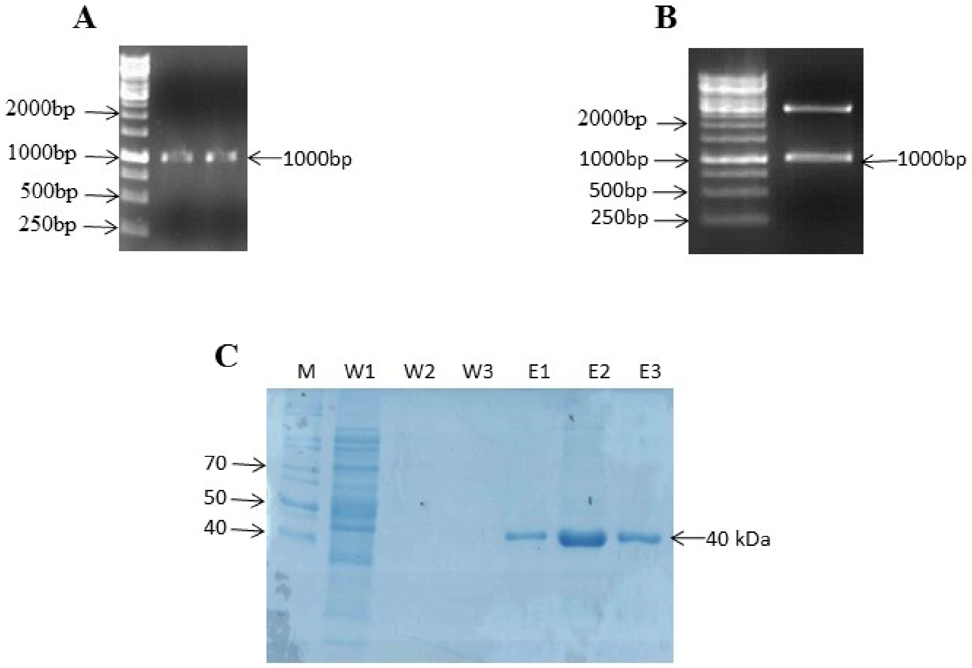

Figure S1: A. PCR Amplification: 1% Agarose gel showing PCR amplification of *Rv3131*. Lane 1 denotes 1 kb ladder; lanes 2-3 denote ~1000bp product of amplified gene *Rv3131*. **B. Clone confirmation:** 1% Agarose gel showing double digestion of *Rv3131*. Lane 1 denotes 1 kb ladder; lane 2 denotes double digestion of *Rv3131* with BamH1 and Hind III. **C. Purification of recombinant protein:** His tagged Rv3131 was purified by Talon metal affinity chromatography to near homogeneity. Lane 1 represents marker, lanes 2-4 represent washes, lanes 5 – 7 represent the eluted Rv3131 (~40kDa).

---

*Corresponding author: Niyaz Ahmed, Department of Biotechnology and Bioinformatics, University of Hyderabad, Gachibowli, Hyderabad – 500046, India. Tel: + 91 40 66794585 Email: ahmed.nizi@gmail.com
